# Safety and effectiveness of bariatric surgery: Roux-en-y gastric bypass is superior to gastric banding in the management of morbidly obese patients: a reply to the response by Bhoyrul et al

**DOI:** 10.1186/1754-9493-3-20

**Published:** 2009-08-19

**Authors:** Ulrich Guller, Lazar V Klein, John A Hagen

**Affiliations:** 1 Center for Excellence in Bariatric Surgery, Humber River Regional Hospital Finch Site, University of Toronto, Department of Surgery, Toronto, ON, M3N 1N1, Canada; 2 Department of Surgery, Division of Visceral Surgery and Transplantation, Inselspital, University of Bern, 3010 Bern, Switzerland

## Abstract

**Background:**

We have read the letter by Bhoyrul et al. in response to our recently published article "*Safety and effectiveness of bariatric surgery: Roux-en-Y gastric bypass is superior to gastric banding in the management of morbidly obese patients"*. We strongly disagree with the content of the letter.

**Results and discussion:**

Bhoyrul et al. base their letter mostly on low level evidence such as single-institutional case series (level IV evidence) and expert opinion (level V evidence). Surprisingly, they do not comment on the randomized controlled trial, which clearly favours gastric bypass over gastric banding.

**Conclusion:**

The letter by Bhoyrul et al. is based on low level evidence and is itself biased, unsubstantiated, and not supported by the current literature.

## Letter

We have read the letter by Bhoyrul et al. [1] in response to our recently published article ***Safety and effectiveness of bariatric surgery: Roux-en-Y gastric bypass is superior to gastric banding in the management of morbidly obese patients ***[2]. We strongly disagree with the content of the letter. There continues to be much often heated debate regarding the potential benefits of laparoscopic banding versus Roux-en-Y bypass. Not infrequently, personal interests and financial incentives bias surgeons considerably regarding the effectiveness and perceived advantages of these procedures. However, it is of cardinal importance that surgeons and other health care professionals unemotionally base their opinion on high-level evidence such as randomized controlled trials and well-performed systematic reviews.

It is a fact that the available high level evidence clearly favours gastric bypass over gastric banding. The only currently available randomized controlled trial comparing laparoscopic banding versus laparoscopic Roux-en-Y bypass [3] convincingly demonstrates that patients undergoing Roux-en-Y bypass had significantly greater weight loss compared to the banding patients. Conversely, weight loss failure was significantly more prevalent in the gastric banding group. This difference in favour of the laparoscopic gastric bypass group was not only statistically significant but also clinically relevant.

Similarly, the vast majority of comparative studies favour gastric bypass. A recently published, well-researched and well-performed systematic review included 14 studies comparing Roux-en-Y bypass versus gastric banding [4]. This investigation showed that excess body weight loss was consistently and statistically significantly better in patients undergoing laparoscopic Roux-en-Y bypass. Furthermore, the rate of resolution of comorbid diseases such as diabetes, arterial hypertension, dyslipidemia, sleep apnea syndrome, and osteoarthritis was consistently better in the bypass group. Finally, patient satisfaction was significantly higher in the bypass group. Based on these results, the authors of this systematic review concluded that laparoscopic gastric bypass should be the primary bariatric procedure in the management of morbid obesity.

It is surprising and unfortunate that the letter to the Editor by Bhoyrul et al. is not supported by any high-level scientific evidence whatsoever. Conversely, the authors base their opinion mostly on low level evidence such as single-institutional case series (level IV evidence) and expert opinion (level V evidence) [5]. Most surprisingly, the authors do not comment on the only randomized controlled trial comparing laparoscopic Roux-en-Y bypass versus gastric banding, a trial, which clearly favoured gastric bypass. Their letter is itself biased, unsubstantiated, and not supported by the current literature.

## Competing interests

The authors declare that they have no competing interests.

## Authors' contributions

UG drafted the letter. JAH and LVK provided impor tant input. All authors read and approved the final manuscript.
